# Impact of a Web-Based Electronic Health Record on Behavioral Health Service Delivery for Children and Adolescents: Randomized Controlled Trial

**DOI:** 10.2196/10197

**Published:** 2018-06-14

**Authors:** Eric J Bruns, Alyssa N Hook, Elizabeth M Parker, Isabella Esposito, April Sather, Ryan M Parigoris, Aaron R Lyon, Kelly L Hyde

**Affiliations:** ^1^ Department of Psychiatry and Behavioral Sciences University of Washington School of Medicine Seattle, WA United States; ^2^ FidelityEHR Santa Fe, NM United States

**Keywords:** mental health, medical informatics, electronic health records, child, adolescent, integrated care, care coordination

## Abstract

**Background:**

Electronic health records (EHRs) have been widely proposed as a mechanism for improving health care quality. However, rigorous research on the impact of EHR systems on behavioral health service delivery is scant, especially for children and adolescents.

**Objective:**

The current study evaluated the usability of an EHR developed to support the implementation of the Wraparound care coordination model for children and youth with complex behavioral health needs, and impact of the EHR on service processes, fidelity, and proximal outcomes.

**Methods:**

Thirty-four Wraparound facilitators working in two programs in two states were randomized to either use the new EHR (19/34, 56%) or to continue to implement Wraparound services as usual (SAU) using paper-based documentation (15/34, 44%). Key functions of the EHR included standard fields such as youth and family information, diagnoses, assessment data, and progress notes. In addition, there was the maintenance of a coordinated plan of care, progress measurement on strategies and services, communication among team members, and reporting on services, expenditures, and outcomes. All children and youth referred to services for eight months (N=211) were eligible for the study. After excluding those who were ineligible (69/211, 33%) and who declined to participate (59/211, 28%), a total of 83/211 (39%) children and youth were enrolled in the study with 49/211 (23%) in the EHR condition and 34/211 (16%) in the SAU condition. Facilitators serving these youth and families and their supervisors completed measures of EHR usability and appropriateness, supervision processes and activities, work satisfaction, and use of and attitudes toward standardized assessments. Data from facilitators were collected by web survey and, where necessary, by phone interviews. Parents and caregivers completed measures via phone interviews. Related to fidelity and quality of behavioral health care, including Wraparound team climate, working alliance with providers, fidelity to the Wraparound model, and satisfaction with services.

**Results:**

EHR-assigned facilitators from both sites demonstrated the robust use of the system. Facilitators in the EHR group reported spending significantly more time reviewing client progress (*P*=.03) in supervision, and less time overall sending reminders to youth/families (*P*=.04). A trend toward less time on administrative tasks (*P*=.098) in supervision was also found. Facilitators in both groups reported significantly increased use of measurement-based care strategies overall, which may reflect cross-group contamination (given that randomization of staff to the EHR occurred within agencies and supervisors supervised both types of staff). Although not significant at *P*<.05, there was a trend (*P*=.10) toward caregivers in the EHR group reporting poorer shared agreement on tasks on the measure of working alliance with providers. No other significant between-group differences were found.

**Conclusions:**

Results support the proposal that use of EHR systems can promote the use of client progress data and promote efficiency; however, there was little evidence of any impact (positive or negative) on overall service quality, fidelity, or client satisfaction. The field of children’s behavioral health services would benefit from additional research on EHR systems using designs that include larger sample sizes and longer follow-up periods.

**Trial Registration:**

ClinicalTrials.gov NCT02421874; https://clinicaltrials.gov/ct2/show/NCT02421874 (Archived by WebCite at http://www.webcitation.org/6yyGPJ3NA)

## Introduction

### Background

Electronic health record (EHR) systems are a type of health information technology (HIT) that has been widely proposed as a mechanism for improving the quality and positive impact of health care services [1-4]. Research suggests that a well-implemented and fully-integrated EHR systems can promote complete record-keeping and more efficient access to documentation, facilitating information sharing and better coordination of care [1,5,6]. Other proposed, but less well-validated, benefits of EHR systems include: (1) facilitating the use of standardized assessments that can promote progress monitoring, (2) better linkage to evidence-based interventions, (3) more effective communication between providers and supervisors, and (4) use of data to promote quality improvement and research [7].

Given the potential benefits, the use of EHR technology in healthcare has been a high policy priority for well over a decade, as evidenced by enabling legislation such as the Health Information Technology for Economic and Clinical Health (HITECH) Act [8], which authorized incentive payments through Medicare and Medicaid to eligible providers. Accordingly, research on EHRs in general healthcare has proliferated over the past decade. Research has examined rates of uptake of EHR systems and related HIT across healthcare settings [9-11], illuminated factors related to adoption and perceived usability [6,9], and enumerated barriers, challenges, and strategies to promote implementation [4-7,12,13]. Importantly, research has also examined impacts of EHR adoption, with comprehensive reviews showing a mix of positive, negative, and null outcomes. In general, studies have found structural and process benefits, such as productivity and work practices, but less impact on clinical outcomes [14-16].

### Electronic Health Records in Behavioral Healthcare

In contrast to general healthcare, EHRs in behavioral health (ie, substance abuse and mental health services) has lagged substantially in both policy and research. Behavioral health providers were excluded from incentive programs such as those promoted by HITECH, rendering most behavioral healthcare providers unable to qualify for incentive payments [17]. Thus, it is not surprising that utilization of EHR systems in specialized behavioral health settings and addiction treatment centers is still quite limited [14], with a 2012 study finding full EHR adoption in only about 20 percent of 505 behavioral health organizations [11].

Research on behavioral health information technology in general—and EHR implementation and impacts specifically—is also sparse by comparison to general healthcare [18,19]. Research that does exist has tended to find parallels to general healthcare. For example, the most commonly implemented EHR components for behavioral healthcare include maintaining documentation on clients and services provided, billing, scheduling, and clinic-wide reporting [5,6,17]. Functions such as information exchange, progress monitoring, and quality assurance—components that are arguably most likely to directly impact the content and quality of services delivered—were endorsed less frequently [14,20].

Barriers to behavioral health providers’ EHR adoption and implementation also have been found to parallel those for general healthcare providers, with financial barriers related to procuring and maintaining EHRs most prominent, but also including issues related to technical support, lack of enthusiasm among providers, and the time and effort required for training and implementation [14,19,21,22]. Unique concerns have also been raised, such as poor alignment with existing behavioral health workflows, lack of fit between the types of information maintained by behavioral health providers (which may be more narrative) and typical EHR structures and functions (which are often more quantitative and categorical), and negative impacts on provider–client communication that may impede therapeutic alliance [14,23-25]. Although some studies have found that behavioral health providers prefer using EHR systems over paper records [25], others have found relatively low rates of satisfaction with the usability and helpfulness of EHR systems and the need for “work arounds” [22,26-28]. For example, a survey of 46 children’s behavioral health providers conducted by our research team (personal communication with Coldiron, Hensley, and Hadfield, 2018) found a mean (SD) System Usability Scale (SUS) [29] score of only 48.4 (22.7) for the EHRs being used in organizations, well below the cut-off for acceptable (mean score of 65) or even “marginal” (mean score of 50) [30].

In sum, EHR systems continue to be promoted in behavioral healthcare as a potential means of improving practice efficiency and effectiveness. However, behavioral health-focused EHR systems lag substantially behind those for general healthcare in enabling policy and subsequent adoption, and behavioral health providers tend to be more skeptical of benefits than healthcare providers. Research is also scant by comparison, with most research to date focused on rates of adoption and barriers to EHR use. One recent review concluded that “comparative studies exploring EHR implementation within behavioral health settings are currently absent in the literature” [25]. While a few studies have suggested EHRs may promote better coordination among primary care and behavioral health providers [9,18], little research is available to shed light on EHR systems’ impact on practice, process, and client outcomes.

### Electronic Health Record Systems and Care Coordination

One area of behavioral healthcare that may especially benefit from an expansion of the EHR research base is care coordination for individuals with multiple and complex behavioral health needs. Effective care coordination requires a range of practitioner communication, service provision, and administrative activities with the potential to be facilitated by technology. Examples include: sharing of information among providers, accessibility of records by clients and their families, such as in personalized health records [10], access to a diverse provider registry, billing for multiple services and strategies, and cost and outcomes monitoring at the client, program, and system levels [31,32].

Research is now emerging that demonstrates EHRs’ potential for positive impact within coordinated care models. For example, Matiz and colleagues [33] found that enhancements to the EHR that added a care plan template were associated with a fourfold increase in care plan use. King and colleagues found that EHR use was associated with physicians’ adherence to research-based care coordination processes [34]. And Hsiao et al found that physicians using EHR were more likely to receive patient information needed for care coordination than those who did not [35].

While the above studies underscore the potential for EHR systems and other types of HIT to facilitate implementation of effective care coordination, none focused on behavioral healthcare, and none used an experimental design. Overall, despite the potential implications for decision-making among providers, managed care entities, and state behavioral health authorities, research is limited regarding how EHR adoption may affect implementation quality, client satisfaction, and adherence to defined practice models.

### The Current Study

In the current study, we examined usability, and short-term impacts of an EHR developed to support the implementation of care coordination for children and youth with complex behavioral health needs and their families using the Wraparound process [36,37]. This EHR software was found in development studies to have adequate usability under controlled conditions [32]. In this study, we conducted a randomized pilot test of the EHR, assigning Wraparound facilitators working in two provider organizations across two states to either use the new EHR (19/34, 56%) or continue to implement Wraparound services as usual (SAU) using paper-based documentation (15/34, 44%). In our research we sought to determine the following: (1) to understand providers’ perceptions of the EHR's feasibility, acceptability, and contextual appropriateness in the “real world” of implementing Wraparound care coordination, and (2) comparing Wraparound facilitators randomly assigned to use the EHR versus paper-based SAU, determine how EHR implementation affected relevant work practices and service processes, such as supervision, fidelity to the Wraparound practice model, collection and use of progress data, teamwork and alliance, and parent satisfaction with care.

## Methods

### Overview of Study Design

The study was conducted in two sites. Site 1 was a Wraparound agency located in a diverse, largely rural region of a Southeastern US state. Site 2 was a regional mental health center providing Wraparound and other services in a small, predominantly white city and surrounding region in a Midwestern US state.

The study employed a blocked randomized control design with Wraparound facilitators (typically Bachelor’s or Master’s level mental health practitioners). Wraparound facilitators (also care coordinators) were randomly assigned to two conditions, EHR or SAU. A pool of 34 (29 in Site 1 and five in Site 2) randomized facilitators were stratified by the two sites and five supervisors (three in Site 1 and two in Site 2) to balance clustering effects. Randomization was conducted by the independent academic partner at the University of Washington.

All facilitators continued to provide Wraparound care coordination as they did before the study, with one exception: Facilitators assigned to the EHR condition were trained and supported to use an online EHR software package (see below for details). Facilitators not assigned to use the EHR continued to provide SAU. All supervisors were also trained to use the EHR and were encouraged to use the system when supervising facilitators in the EHR group. However, it is important to note that all supervisors were asked to supervise facilitators in both study groups.

### Participants

#### Youth

To be eligible for the study, children and youth had to be between 5 and 18 years old and experiencing serious emotional and behavioral disturbance, defined as having a mental health diagnosis as designated in the Diagnostic and Statistical Manual of Mental Disorders, 5^th^ Edition [38] and functional impairment that “substantially interferes with or limits the child from developing social, behavioral, cognitive, communicative or adaptive skills or his activities relating to family, school or community.” Youth in foster care were not eligible for the study due to issues of obtaining consent for youth in state custody. Youth in multiple sibling groups referred for services were also not eligible due to clustering effects and subsequent difficulty in interpreting results for such families.

**Figure 1:**
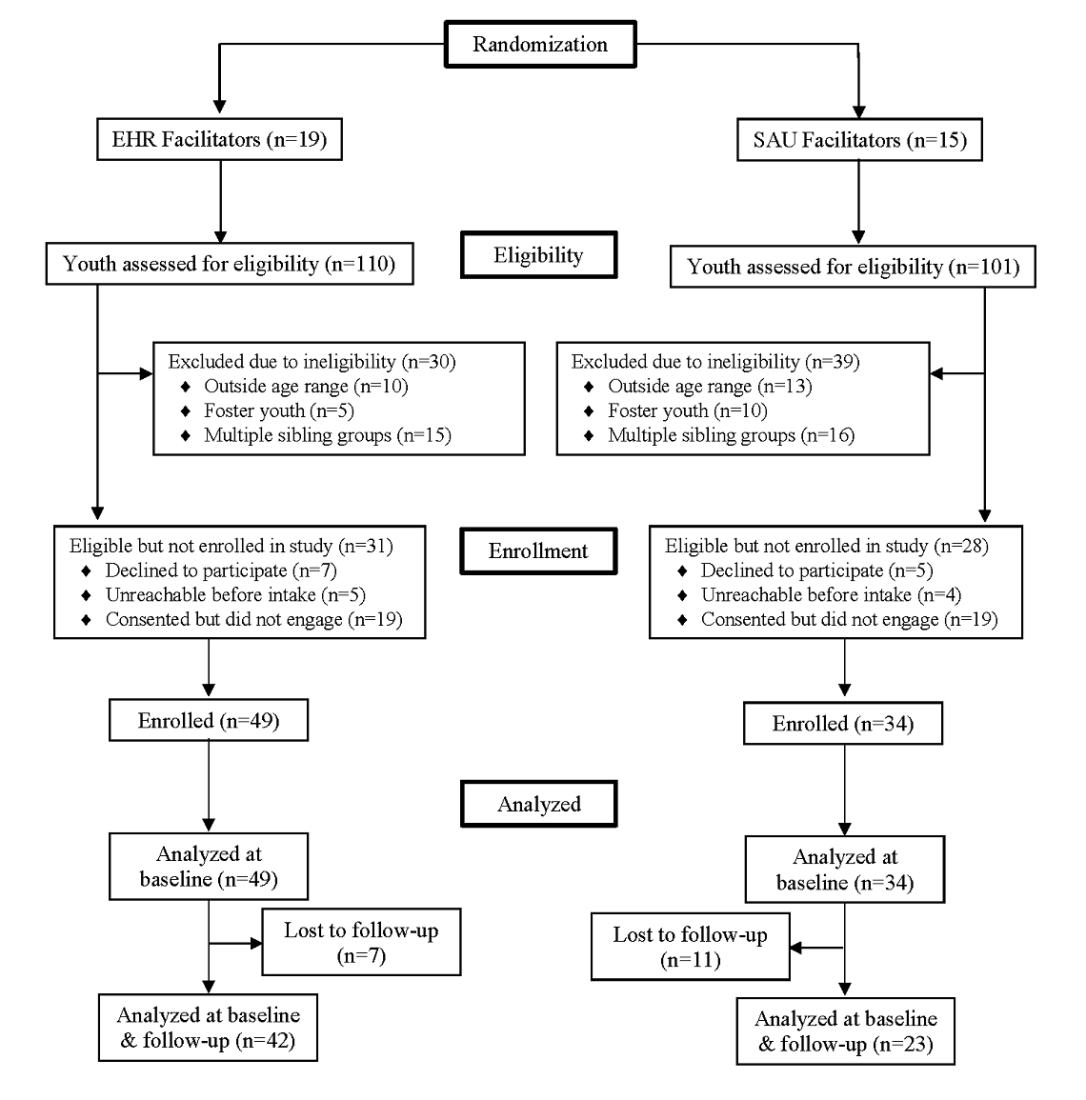
Participant flow through the study.

The study was initiated in November 2015 in Site 1 and January 2016 in Site 2. All 34 facilitators and five supervisors in both sites consented to participate. For eight months after study inception, 211 children and youth enrolled in Wraparound in the two sites were referred to the study. Of those, 69/211 (33%) were found to be ineligible (31/69 [45%] due to being members of sibling groups, 15/69 [22%] due to being foster youth, and 23/69 [33%] due to being out of the age range). Of those remaining, 12/211 (6%) declined to participate, 38/211 (18%) consented to be contacted but did not respond to outreach from the research team, and 9/211 (4%) consented to be in the study but were not responsive to requests to conduct an intake interview. Thus, 83/211 (39%) children and youth and their caregivers were formally enrolled in the study, 49/83 (59%) served by facilitators in the EHR condition and 34/83 (41%) served by facilitators in the SAU condition. Of these, 18/83 (22%) were lost to data collection follow up leaving a final sample of 65/83 (78%) children and youth for whom longitudinal data were available, 42/65 (65%) in the EHR group and 23/65 (35%) in the SAU group. A detailed Consolidated Standards of Reporting Trials (CONSORT) diagram is provided in Figure 1.

As shown in Table 1, the group of children and youth on which analyses were conducted was majority male (54/83, 65%), with a mean age of 11.4 (SD 3.73) years. Approximately half (42/83, 51%) were from a racial or ethnic minority group (African American (37/83, 45%), mixed race (5/84, 6%), and 1/83 (1%) of Hispanic ethnicity). The most common Axis I diagnoses across children and youth were attention disorders (33/83, 40%), mood disorders (20/83, 24%), oppositional and conduct disorders (11/83, 13 %), and anxiety disorders including PTSD (11/83, 13%).

**Table 1:** Baseline characteristics of children and youth, caregivers, and facilitators by study group.

Characteristics				EHR^a^			SAU^b^			Total
**Youth, n**				49			34			83
	Age in years, mean (SD)			11.35 (3.69)		11.56 (3.85)			11.43 (3.73)	
	Female, n (%)			18 (36.73)		11 (32.35)			29 (34.94)	
	**Race, n (%)**									
		African American	19 (38.78)		18 (52.94)			37 (44.58)		
		White	27 (55.10)		14 (41.18)			41 (49.40)		
		Mixed	3 (6.12)		2 (5.88)			5 (6.02)		
	Repeated a grade, n (%)			19 (40.43)		12 (35.29)			31. (38.27)	
	Ever been in foster care, n (%)			9 (18.37)		6 (17.65)			15 (18.07)	
	Brief Problem Checklist-Total problem score			14.02 (4.44)		13.56 (4.78)			13.83 (4.56)	
	Strengths and Difficulties Questionnaire-Total Score			21.26 (7.22)		21.47 (5.95)			21.94 (6.71)	
**Caregiver, n**				49			34			83
	Age in years, mean (SD)			38.49 (8.47)		39.32 (11.19)			38.83 (8.47)	
	Female, n (%)			45 (91.84)		33 (97.1)			78 (93.98)	
	**Race, n (%)**									
		African American	18 (36.73)		17 (50)			35 (42.17)		
		White	30 (61.22)		17 (50)			47 (56.63)		
		Other	1 (1.20)		0			1 (2.04)		
	**Adjusted gross income (US), n (%)**									
		< $19,000	26 (54.17)		23 (39.70)			49 (60.49)		
		$20,000-$39,000	15 (31.25)		7 (21.21)			22 (27.16)		
		>$40,000	7 (14.58)		3 (9.09)			10 (12.35)		
	**Relationship to youth, n (%)**									
		Biological parent	37 (75.51)		26 (76.47)			63 (75.90)		
		Adoptive parent	4 (8.16)		1 (2.94)			5 (6.02)		
		Grandparent	3 (6.12)		5 (14.17)			8 (9.64)		
		Other	5 (10.20)		2 (5.88)			7 (8.43)		
**Facilitator, n**				18			13			31
	Female, n (%)			11 (61.1)		11 (84.6)			22 (71)	
	**Race, n (%)**									
		African American	5 (27.8)		2 (15.4)			7 (22.6)		
		White	12 (66.7)		10 (76.9)			22 (71)		
		Hispanic	0		1 (7.7)			1 (3.2)		
		Other	1 (5.6)		0			1 (3.2)		

^a^EHR: electronic health records.

^b^SAU: services as usual.

A total of 29/83 (35%) children and youth had more than one Axis I disorder, and 63/79 (80%) scored in the clinical range for the Total Difficulties Score on the Strengths and Difficulties Questionnaire [39]. As shown in Table 1, there were no significant differences between groups at baseline on any of these measures.

#### Caregivers

Across both groups, 63/83 (76%) of children and youth were cared for by biological parents, 8/83 (10%) by a grandparent, 7/83 (8%) by other individuals (e.g., a family friend), and 5/83 (6%) by adoptive parents. A large majority of caregivers were female (78/8, 94%); 47/83 (57%) were white and 35/83 (42%) were African American. A majority of caregivers had a household income of less than US $19,000 (49/81, 60%). As shown in Table 1, there were no between-group differences on any variables at baseline.

#### Facilitators

Three facilitators were lost to attrition in Site 1 (all before study youth were assigned to them); thus, children and youth in the study were served by a total of 31 Wraparound facilitators, 26/31 (84%) in Site 1 and 5/31 (16%) in Site 2. A majority were female (22/31, 71%) and white 22/31 (71%), while 7/31 (23%) were African-American. As shown in Table 1, there were no differences in demographics between facilitators at baseline.

### Intervention Conditions

#### Electronic Health Record Condition

Facilitators assigned to the EHR condition used an online software system that was developed through a partnership between a university research team and a small behavioral health-focused software developer. In addition to standard EHR fields (eg, youth and family information, diagnoses, assessment data, progress notes), the software maintained information on all elements of the Wraparound team and Wraparound plan in formats that align with the defined practice model for Wraparound care coordination [40,41]. For example, the software is organized via tabs that correspond to the sequence of activities that engage the family and build a plan that serves as the focus of coordinated Wraparound teamwork. Examples of information entered and maintained include the family’s background and the reason for referral, youth and family strengths, a team mission statement, and priority need statements in the family’s own words. Each need statement is connected to specific strategies and one or more outcomes statements on which data must be entered over time. If a strategy is a billable service, the facilitator can enter the service, service provider information, and the number of units authorized.

Other functions supported by the EHR system include individualized permission levels that allow for the sharing of information among youth and families, providers, and other team members. As such, caregivers and youth had access to certain records within the system, such as meeting schedules, plans of care, and progress monitoring dashboards. Reporting functions include individual youth-, supervisor-, and administrator-level data aggregation and reporting on services, expenditures, and outcomes. Facilitator workflow is supported by a hyperlinked Task List that tracks the completion of necessary care coordination steps and tasks as well as completion of required fields and elements of the Wraparound plan. The EHR also sends system-generated emails that obtain electronic signatures and automated reminders for upcoming meetings. Finally, the system promotes outcomes monitoring and feedback via collection of data on progress toward youth and family needs statements (eg, on a 0-10 scale). Brief process (eg, connection to professional helpers and social supports) and standardized outcomes measures are also incorporated into the system.

Although all efforts on the part of Wraparound care coordinators were completed within the EHR system, documentation by other involved health professionals (eg, primary or specialty care physicians, child welfare case workers, mental health therapists) was not completed within the same EHR system. Evaluation reports, medical records, and other documentation can, however, be uploaded to the record via secure upload. See work published by Bruns and colleagues [32] for more details on the system.

Facilitators in the EHR group were trained on and supported to use the software via a sequence of activities that included:(1) an online training; (2) a two-day in-person training from the software developer’s training team; and (3) monthly web-based check-in calls with five small cohorts of facilitators organized by supervisor. For two months after initial training (but before enrollment of study families), EHR-assigned facilitators were supported to continue learning the functions of the EHR with the two youth/families on their caseloads who were most recently enrolled in services. EHR users also had the availability of help desk support. The research team sent regular reports of EHR system use and data completeness for study enrolled families to facilitators and supervisors to help encourage full use of the system.

#### Services as Usual Condition

Facilitators in the control group completed research measures as described below, but did not participate in the training or use of the EHR. Rather, SAU-assigned facilitators maintained documentation, as usual, involving traditional paper case files. For facilitators in the SAU group, intake paperwork, progress notes, Wraparound plans, meeting minutes, and assessments all continued to be typed and hand-written and stored in a paper file and/or Excel files. Supervisors of SAU facilitators continued to review information on family needs, plans, and progress using paper and Excel files in their management and supervision.

### Measures

#### Electronic Health Record System Activity

The research team monitored use of the software by EHR-assigned facilitators and reviewed activity logs by the facilitator in monthly consultation. The research team also compiled and fed these data back in initial months of the study to ensure the system was being used as intended by EHR group members. The activity monitor recorded each movement the user made within the system (ie, “Visited Custom Assessment Report Page” or, “Visited Add/Edit User Page”) to capture how facilitators were utilizing the system. These data were then aggregated into categories (ie, “Maintaining Service Notes” or, “Updating & Developing the Plan of Care”) to assess the percentage of time users were spending on each type of function in the EHR.

#### Demographic Information

The *Family Information Form* obtains data on youth and family demographics (eg, age; gender, and race of children/youth and caregivers; family income), diagnosis, and other information related to the family’s history and home composition. The survey was administered to caregivers at baseline.

#### Provider Perceptions of Electronic Health Records

All supervisors and EHR-assigned facilitators completed two measures of EHR usability, acceptability, feasibility, and appropriateness six months after initiation of the study and training on the system. *The System Usability Scale (SUS)* is a widely used, 10-item measure of perceptions of the usability of a technology system developed by Brooke [42,43]. Items such as, “I thought the system was easy to use,” and, “I felt very confident using the system” are rated on a five-point Likert scale. Resulting total scores range from 1-100. Scores below 50 indicate unacceptable usability, 50-70 indicate marginal usability, and greater than 70 indicate acceptable usability [29,42]. This scale is well-validated and has been found to have high inter-rater and test-retest reliability, excellent internal consistency (alpha=.91) [44], and significant associations with alternative usability evaluation approaches [45].

The *System Acceptability and Appropriateness Scale (SAAS)* is an 11-item measure that evaluates HIT acceptability, utility and fit with service context. Items such as “How relevant is the technology to your client population?” are rated on a 1 (Not at All) to 5 (Extremely) point Likert scale and result in two subscale scores: Acceptability and Appropriateness. The SAAS was adapted from existing measures of intervention and HIT acceptability, including the Usage Rating Profile Intervention [46], Treatment Acceptability Rating Form-Revised (TARF-R) [47], and Intervention Rating Profile-15 [48]. Versions of the SAAS have been shown to possess acceptable technical adequacy (alpha>.70) and criterion-related validity [49].

#### Provider Workflow and Behaviors

Four measures focused on provider workflow and behaviors. Two measures focused on supervision practices, one on facilitator attitudes toward standardized assessments, and one on facilitator behaviors related to measurement-based care.

The *Supervision Process Questionnaire (SPQ)* asked supervisors and facilitators to evaluate the percentage of time spent during supervision in nine different areas (eg, crisis assessment, client progress review, case conceptualization). Subscale or total scores were not calculated; instead, data were analyzed at the individual item level to evaluate between-group differences in supervision foci. Preliminary studies have found adequate interrater reliability [50].

The *Brief Supervision Practice Checklist-Adapted (BSPC)* is an eight-item survey administered to supervisors and facilitators that collects information on types of supervision practices and asks individuals in both roles to rate on a five-point scale (from Never to Almost Always) the degree to which different types of supervision practices are provided (eg, “supervisor discussed techniques to encourage family engagement;” and “supervisor reviewed youth and family progress”). Originally developed by Dorsey and colleagues [51] for supervision of clinicians practicing individual therapy, the measure was revised to better align with Wraparound care coordination. Formal reliability and validity have not been reported; however, internal consistency for the current sample was found to be good (alpha=.93). Total scores were calculated for BSPC items, given that, unlike the SPQ, all items are proposed to evaluate a latent variable focused on effective Wraparound supervision. The SPQ and BSPC were administered to supervisors and facilitators in both groups at the initiation of the study (before training on the EHR), and six months later.

The *Attitudes Toward Standardized Assessment Scale (ASA)* is a 22-item measure of practitioner perceptions and attitudes about using standardized assessments in clinical practice. Items are scored on a 1 (Strongly Disagree) to 5 (Strongly Agree) scale and yield three subscales with adequate or better reliabilities: Benefit over Clinical Judgment, Psychometric Quality, and Practicality (alpha=.75) [52]. Ratings have been associated with a greater likelihood of standardized assessment use. Facilitators in both groups completed the ASA at baseline and six-month time points.

The *Current Assessment Practice Evaluation-Revised (CAPER)* is a 10-item measure that assesses practitioners’ self-ratings of behaviors related to measurement-based care (MBC) across different phases of intervention (eg, at intake, ongoing during termination, discharge). As described in a recently submitted paper by Lyon and colleagues, CAPER subscales demonstrated good reliability as well as convergent and divergent validity with clinician attitudes about MBC in the expected directions (personal communication by Aaron Lyon, 2017). Facilitators responded to items such as “In the last two weeks, for how many youth/families did you administer a standardized assessment measure?” and “…for how many families did you systematically track an individualized outcome variable?” Facilitators in both groups completed the CAPER every other week for eight months, for a total of 16 biweekly surveys.

#### Wraparound Implementation and Service Process

Wraparound Implementation and Service Process was evaluated using three measures. The *Team Climate Inventory, short version (TCI)* is a 14-item survey that evaluates five relevant aspects of health care teamwork (Shared Vision, Participation Safety, Support for Innovation, Task Orientation, Interaction Frequency) using a five-point Likert scale. The scale has extensive support for reliability and factor structure; and validity is found in association with healthcare quality, patient satisfaction, and outcomes, including alpha coefficients of the subscales ranging from 0.73-0.80 [53].

The *Wraparound Fidelity Index, Brief Version (WFI-EZ)* is a widely-used, reliable and valid self-report measure of fidelity to the Wraparound process, based on the original Wraparound Fidelity Index, version 4 [54]. Items in the 25-item fidelity section of the measure are rated on the Likert scale from 1 (Strongly Disagree) to 5 (Strongly Agree). The WFI-EZ yields scores for five theory- and research-based Wraparound practice domains (eg, Team-based, Outcomes-based, Family-driven) and a Total Score. Internal consistency for all items has been found to be good (alpha=.89; personal communication with Ryan Parigoris, 2017). Evidence for validity includes differentiation among programs using a method of known groups approach as well as significant correlations between total fidelity scores and alternate measures of fidelity (personal communication with Ryan Parigoris, 2017) [55].

The *Working Alliance Inventory (WAI)* measures alliance between clinicians and clients on three domains: bond, goals, and tasks [56]. Based on the WAI short form, this measure was revised to reflect the alliance between Wraparound facilitators and families. Items are rated on a seven-point Likert scale, with response options ranging from Never to Always. The measure results in an overall alliance score, as well as three subscale scores tied to the domains. Reliability has been found to be good for the client form [57], and adequate for provider versions [56].

Caregivers completed the TCI, WFI-EZ, and WAI four months after entry to Wraparound services.

#### Client Satisfaction

The *Client Satisfaction Questionnaire (CSQ)* is a widely used, well-validated measure of satisfaction with behavioral health services [58]. Items such as “How would you rate the quality of service your child received?” are rated on a four-point Likert scale ranging from Poor to Excellent. For this study, the eight-item short form (CSQ-8) was used, which has an internal consistency of .93 [59]. The CSQ-8 was administered to caregivers four months after entry to Wraparound services.

#### Facilitator Satisfaction

The *Therapist Satisfaction Index (TSI)* is a 14-item self-report measure to assess practitioners’ affinity for the intervention being used, perceived effectiveness, capacity for individualization and flexibility, and applicability to children and youth they work with. Items such as “The caregivers I work with seem to like the Wraparound approach” are rated on a five-point Likert scale from Strongly Disagree to Strongly Agree. Cronbach’s alpha for the total score has been found to be .83 [60]. A version with items revised to be appropriate for Wraparound was administered to facilitators at baseline and six months after study initiation.

### Procedures

The study protocol was approved by the institutional review board at the University of Washington. Provider staff (supervisors and facilitators) were consented by the research coordinator after an on-site study introduction. Provider staff in both conditions completed EHR perception surveys and workflow and provider behavior instruments six months after training on the EHR. Enrollment of children, youth, and caregivers (and baseline interview completion) began two months after initial training. As described above, facilitators completed measures related to implementation specific to each enrolled youth and family four months after the child or youth was enrolled in services. Facilitators also completed a brief online survey about their use of measurement-based care (via the CAPER) bi-weekly for the duration of the study.

Intake coordinators at both study locations assigned all children and youth newly enrolled in the two Wraparound programs a study identification number and assessed them for eligibility. If determined to be eligible, Wraparound facilitators presented eligible youths’ parent or guardian with information about the study and sought to obtain consent to be contacted by the research team. After consent to contact was obtained, a member of the research team contacted the parent/guardian via phone and further explained the parameters of the research study, and formally enrolled those who agreed to participate. Interviews were conducted by a research assistant via phone at baseline and four months.

### Data Analysis

Equivalence of groups at baseline was assessed using t-tests and chi-square tests. Differences between EHR and SAU in provider workflow, implementation and service processes, and client/facilitator satisfaction were examined using t-tests, and hierarchical linear models were also conducted to account for the nested nature of the data. Facilitators (level 1) were nested within supervisors (level 2), who were nested within site (level 3), or caregivers (level 1) were nested within facilitator (level 2), who were nested within site (level 3). To explore the impact of nesting, intraclass correlation coefficients (ICCs) at the supervisor and site levels were examined, both of which were very low (<0.05) for all major outcomes. Therefore, two-level hierarchical linear models with a random intercept for a site were run. To account for missing data, which ranged between 17/83 (20%) to 20/83 (24%), multivariate normal multiple imputations were used with 100 imputations. Auxiliary variables were included to aid the imputation. These analyses were conducted using Stata Version 13.1.

Longitudinal outcomes were tested through two-level growth curve models using HLM 7.0 [61] with observations/time (level 1) nested within facilitators (level 2). The data were also nested by site (level 3), but due to the low ICCs, a dummy variable was created and included in the model. Estimated scores and rates of change over time for the outcome variables were modeled. Random intercepts for facilitator were included and random slopes for observations/time were examined and retained when statistically significant. Data were modeled using full maximum likelihood estimation.

Although a large number of comparisons were made, we chose not to use a correction primarily because it would result in extremely small *P* values (or alphas). All tests were planned *a-priori* so we looked for consistency and examined patterns among the results. Also, because of small sample sizes and the exploratory nature of this study, we flagged results that trended toward significance (between-group differences at *P*<.10 level) for inclusion in the interpretation of results.

## Results

### Group Comparability

The EHR and SAU groups were compared at baseline on several demographic variables (see Table 1). As shown, there were no significant differences between groups on any variables, including total scores on two commonly used measures of child emotional and behavioral functioning, the Brief Problem Checklist [62] and the Strengths and Difficulties Questionnaire [39].

### EHR System Activity

Table 2 presents a summary of EHR activity by facilitators in each site for months 1 and 2 when activity was recorded and fed back during EHR consultation with the research team. As shown, EHR facilitators from both sites demonstrated robust use of the system. Facilitators in Site 1 demonstrated a greater mean number of clicks during the first month at 1,473 (SD 61.45) and second month at 1,060 (SD 58.90) than facilitators from Site 2 with 866 (SD 26.43) in month 1 and 612 (SD 16.91) in month 2. Use by facilitators in both sites was greater in month 1, during which it was necessary to transfer data from paper records for enrolled families. During the second month of use, nearly two-thirds (642/1060, 61%) of the clicks for facilitators in Site 1 were used in communicating with the team or updating and developing the plan of care. In contrast, a larger proportion of clicks for facilitators from Site 2 (395/612, 65%) were used in the system managing information and updating/maintaining service notes. Such differences were attributed to different organizational priorities and approaches to implementing Wraparound.

### Provider Perceptions

Results from measures focused on practitioner perspectives on the EHR including the SUS and SAAS are summarized in Table 3. Scores on the individual items of the SUS ranged from 1.6-2.7, with a total average score of 54.72 (range 30-70.3). Scores on the acceptability subscale ranged from 2.6-3.6 and scores on the appropriateness subscale ranged from 2.9-3.4.

### Provider Workflow and Behaviors

Workflow outcomes were assessed using measures of supervision activity (SPQ and BSPC), use of measurement-based care (CAPER), and attitudes toward standardized assessment (ASA). Results are presented in Table 4. There were no differences between groups on the ASA scale at baseline. Results from the HLM suggested facilitators in the EHR group reported lower scores on the psychometric quality subscale, on average, compared to those in the SAU group. Facilitator reports did not differ by treatment group for the “benefit over clinical judgment” and “practicality” subscales.

**Table 2:** Summary of system activity (number of clicks) by site and time for the first two months of the study.

EHR^a^ function	Site 1 (n=18), mean (%)		Site 2 (n=13), mean (%)	
	Month 1	Month 2	Month 1	Month 2
Communicating with the team	305 (20.7)	431 (40.7)	138 (15.9)	15 (2.4)
Core Assessments	4 (0.3)	25 (2.4)	25 (2.8)	34 (5.6)
Maintaining service notes	177 (12.0)	162 (15.3)	129 (14.8)	142 (23.2)
Managing information	324 (22.0)	13 (1.2)	273 (31.5)	253 (41.4)
Updating and developing the Wraparound plan	396 (26.9)	211 (20.0)	136 (15.7)	76 (12.4)
User settings	266 (18.1)	217 (20.5)	167 (19.2)	92 (15.1)
Total	1473 (100)	1060 (100)	866 (100)	612 (100)

^a^EHR: electronic health record.

**Table 3:** Electronic health record software acceptability, appropriateness, and usability at the six-month follow-up (n=18 facilitators).

Variable			Mean (SD)
System Usability Scale - Total usability score (0-100 scale)			54.72 (12.54)
**System Acceptability and Appropriateness (0=lowest to 5=highest)**			
	**Acceptability**		
		Satisfied with current version of the technology	2.83 (0.92)
		Believe technology to be organized/well-constructed	3.11 (1.08)
		Satisfied with content of technology system	2.83 (0.79)
		Satisfied with the technology's overall ease of use	2.61 (0.92)
		Comfortable interacting with the technology	3.56 (0.78)
		The technology is intuitively appealing	3.17 (1.04)
	**Appropriateness**		
		The technology is compatible with agency's mission or service provision mandate	3.39 (0.70)
		The technology is relevant to client population	3.11 (0.90)
		The technology fits with current treatment modality, theoretical orientation, or skill set	3.33 (0.84)
		The technology is compatible with workflow timing	2.94 (0.87)
		The technology fits with overall approach to service delivery and the setting in which care is provided	3.17 (0.87)

**Table 4:** Summary of workflow outcomes by study group.

Variable		EHR^a^ (n=18) facilitators, mean (SD)	SAU^b^ (n=13) facilitators, mean (SD)	*P* value	Intercept coefficient (SE)	Intervention coefficient (SE)	Baseline coefficient (SE)
**Attitudes towards Standardized Assessments**							
	Benefit Over Clinical Judgement	2.77 (0.64)	2.89 (0.33)	.52	2.63 (0.44)^c^	0.18 (0.16)	0.07 (0.15)
	Psychometric Quality	3.35 (0.43)	3.37 (0.36)	.89	1.80 (0.47)^c^	–0.30 (0.10)^d^	0.50 (0.13)^c^
	Practicality	3.21 (0.39)	3.06 (0.31)	.28	2.45 (0.56)^c^	–0.13 (0.12)	0.23 (0.17)
**Brief Supervision Practice Checklist**							
	Supervision Score	3.56 (0.18)	3.32 (0.27)	.45	0.53 (0.47)	0.09 (0.22)	0.76 (0.13)^c^
**Supervision Process Questionnaire**							
	Administrative tasks	8.78 (6.59)	11.53 (7.47)	.29	9.38 (2.26)^c^	–3.36 (2.03)^e^	0.19 (0.15)
	Facilitator personal support	10 (6.89)^f^	3.46 (4.74)^f^	.01	5.22 (1.32)^c^	1.42 (1.84)	0.16 (0.14)
	Reviewing progress toward needs	4.44 (4.08)	5.77 (4)	.38	6.29 (1.62)^c^	3.33 (1.56)^f^	0.11 (0.19)
	Skills coaching and training	12.72 (8.90)	17.23 (9.82)	.19	11.51 (2.78)^c^	–2.32 (2.36)	0.25 (0.13)^f^
	Reviewing plans of care	15.33 (7.14)	16.54 (5.55)	.62	5.16 (3.27)	2.42 (1.99)	0.47 (0.16)^d^
	Crisis assessment management	8.67 (6.71)	6.62 (4.14)	.34	7.58 (1.38)^c^	–1.01 (1.46)	0.08 (0.13)
	Case conceptualization	5.83 (2.96)	5.54 (4.10)	.82	4.30 (1.25)^d^	0.26 (1.12)	0.26 (0.17)
	Youth family engagement	13.06 (2.71)^f^	9.46 (3.82)^f^	.01	6.58 (2.60)^f^	–0.22 (1.63)	0.42 (0.24)^e^
	Natural support engagement	10.28 (4.36)	10.31 (4.75)	.99	11.07 (2.30)^c^	0.49 (1.66)	–0.14 (0.19)
	Support relationship	5.33 (3.24)^f^	9.23 (4.00)^f^	.01	4.74 (3.19)	0.49 (1.39)	0.52 (0.22)^f^
	Facilitator professional role	5.56 (3.38)	4.31 (2.81)	.29	3.14 (0.94)^d^	–0.54 (0.93)	0.43 (0.15)^d^

^a^EHR: electronic health record.

^b^SAU: services as usual.

^c^*P*<.001

^d^*P*<.01

^e^*P*<.10

^f^*P*<.05

As shown, there were no differences between groups on the BSPC score at baseline. However, there were differences on the SPQ. At baseline, facilitators in the EHR group reported greater average scores on the facilitator personal support and youth family engagement subscales compared to facilitators in the SAU group. In comparison, facilitators in the EHR group reported lower average scores on the support relationship subscale compared to the SAU group. In the HLM, reports on the BSPC did not differ when comparing facilitators in the EHR group to the SAU group. On the SPQ, facilitators in the EHR group reported significantly higher scores on the reviewing progress toward needs subscale, compared to those in the SAU group. Additionally, facilitators in the EHR group reported lower scores on the administrative tasks subscale, on average, compared to those in the SAU group (result approached significance, *P<*.10).

### Wraparound Implementation and Service Process

Implementation and fidelity outcomes are presented in Table 5. There were no significant differences between treatment groups across the WAI, TCI, WFI-EZ fidelity total score, or the CSQ (total score) variables at the four-month follow-up. Based on results of HLM, caregiver reports on these measures did not differ significantly for those in the EHR group compared to the SAU group over time. However, a trend (*P*=.10) was found whereby facilitators in the EHR group reported lower scores compared to those in the SAU group on the task subscale of the WAI.

**Table 5:** Summary of caregiver and facilitator-reported implementation and fidelity outcomes by study group.

Variable			EHR^a^, mean (SD)	SAU^b^, mean (SD)	*P* value	Intercept coefficient (SE)	Intervention coefficient (SE)	Baseline coefficient (SE)
**Caregiver**								
	**Team Climate Inventory**							
		Vision	17.10 (3.46)	17.57 (2.31)	.56	17.75 (0.73)^c^	–0.65 (0.88)	N/A^d^
		Participative safety	18 (0.39)	17.30 (3.52)	.38	17.91 (0.83)^c^	–0.75 (0.80)	N/A
		Task orientation	12.98 (2.50)	13.13 (2.16)	.80	13.24 (0.57)^c^	–0.25 (0.65)	N/A
		Support innovation	12.88 (2.67)	13 (2.22)	.86	12.89 (0.72)^c^	–0.17 (0.66)	N/A
	**Working Alliance Inventory**							
		Goal	23.81 (6.39)	24.35 (4.16)	.72	24.39 (1.32)^c^	–0.68 (1.54)	N/A
		Task	23.55 (6.60)^e^	26.04 (3.90)^e^	.1	25.77 (1.59)^c^	–2.66 (1.53)^e^	N/A
		Bond	25.36 (5.55)	26.57 (3.46)	.35	26.23 (1.39)^c^	–1.29 (1.30)	N/A
		Total score	72.71 (17.56)	76.96 (10.23)	.29	76.19 (4.24)^c^	–4.33 (4.22)	N/A
	**WFI-EZ Fidelity**							
		Outcomes	0.74 (0.26)	0.75 (0.12)	.95	0.73 (0.07)^c^	–0.01 (0.06)	N/A
		Teamwork	0.70 (0.21)	0.66 (0.19)	.50	0.65 (0.06)^c^	0.03 (0.05)	N/A
		Natural supports	0.58 (0.20)	0.62 (0.16)	.36	0.63 (0.04)^c^	–0.05 (0.05)	N/A
		Needs	0.72 (0.20)	0.74 (0.12)	.68	0.74 (0.04)^c^	–0.03 (0.05)	N/A
		Strengths	0.80 (0.20)^e^	0.71 (0.14)^e^	.09	0.71 (0.05)^c^	0.07 (0.05)	N/A
	**Parent and Child Satisfaction Scale**							
		Total score	3.40 (0.69)	3.44 (0.59)	.84	3.37 (0.20)^c^	–0.04 (0.18)	N/A
**Facilitator**								
	**Therapist Satisfaction Index**							
		Total score	2.77 (0.64)^e^	2.89 (0.33)^e^	.08	–3.06 (1.66)^e^	3.10 (2.14)	0.99 (0.20)^f^

^a^EHR: electronic health record. n=42 and n=18 for caregiver and facilitator groups, respectively.

^b^SAU: services as usual; multiple imputation (mi) used to handle missing data (mi=100 imputed datasets). n=23 and n=13 for caregiver and facilitator groups, respectively.

^c^*P*<.001

^d^N/A: not applicable.

^e^*P*<.10

^f^*P*<.01

**Table 6:** Time and facilitator level indicators of current practices as reported on the Current Assessment Practice Evaluation Revised.

Variable			Fixed effects coefficient		Standard error		Random effects, SD		Variance
**Administered Standardized Assessment**								
	Intercept	13.26		17.08		N/A^a^		N/A	
	Month (L1)^b^	1.30^c^		0.39		N/A		N/A	
	Intervention (L2)^d,e^	8.87		7.34		N/A		N/A	
	Site (L2)^d,f^	11.77		15		N/A		N/A	
	Facilitator	N/A		N/A		19.48^g^		379.6	
	Month	N/A		N/A		1.62^h^		2.62	
**Given Feedback about Assessment**									
	Intercept	18.27		15.83		N/A		N/A	
	Month (L1)^b^	1.40^g^		0.36		N/A		N/A	
	Intervention (L2)^d,e^	7.68		6.78		N/A		N/A	
	Site (L2)^d,f^	6.29		14.13		N/A		N/A	
	Facilitator	N/A		N/A		17.44^g^		304.2	
	Month	N/A		N/A		1.40^c^		1.95	
**Systematically Tracked Outcome**									
	Intercept	44.69^c^		12.47		N/A		N/A	
	Month (L1)^b^	0.87^h^		0.35		N/A		N/A	
	Intervention (L2)^d, e^	–2.43		6.34		N/A		N/A	
	Site (L2)^d,f^	4.16		12.05		N/A		N/A	
	Facilitator	N/A		N/A		15.79^g^		249.45	
	Month	N/A		N/A		1.16^h^		1.34	
**Given Feedback on Outcome**									
	Intercept	51.13^c^		14.27		N/A		N/A	
	Month (L1)^b^	0.76^h^		0.34		N/A		N/A	
	Intervention (L2)^d,e^	–0.98		6.28		N/A		N/A	
	Site (L2)^d,f^	–2.63		13.71		N/A		N/A	
	Facilitator	N/A		N/A		16.26^g^		264.51	
	Month	N/A		N/A		1.10^h^		1.21	
**Plan of Care Altered Based on Assessment**									
	Intercept	42.95^g^		8.57		N/A		N/A	
	Month (L1)^b^	1.07^g^		0.29		N/A		N/A	
	Intervention (L2)^d,e^	2.61		4.99		N/A		N/A	
	Site (L2)^d,f^	–18.03^c^		5.83		N/A		N/A	
	Facilitator	N/A		N/A		10.14^c^		102.92	
	Month	N/A		N/A		0.95^h^		0.91	
**Assessment Used to Choose Service**									
	Intercept	4.59		15.5		N/A		N/A	
	Month (L1)^b^	0.86^g^		0.24		N/A		N/A	
	Intervention (L2)^d,e^	3.83		5.9		N/A		N/A	
	Site (L2)^d,f^	12.25		13.1		N/A		N/A	
	Facilitator	N/A		N/A		14.51^g^		210.67	
	Month	N/A		N/A		0.62^h^		0.38	
**Sent Reminders**									
	Intercept	69.97^g^		12.86		N/A		N/A	
	Month (L1)^b^	0.47		0.4		N/A		N/A	
	Intervention (L2)^d,e^	–17.28^h^		7.82		N/A		N/A	
	Site (L2)^d,h^	–3.82		8.79		N/A		N/A	
	Facilitator	N/A		N/A		21.59^g^		466.26	
	Month	N/A		N/A		1.62^g^		2.63	

^a^N/A: not applicable.

^b^L1: Level 1 predictor.

^c^*P*<.01

^d^L2: Level 2 predictor.

^e^Intervention: 0=control group (reference), 1=intervention group.

^f^Site: 0=Site 1 (reference) 1=Site 2.

^g^*P*<.001

^h^*P*<.05

Time trends and other results from the growth curve models for the facilitator-completed CAPER are found in Table 6. Significant linear time trends were found for six items, with increasing proportions of facilitators reporting administering standardized assessments, giving feedback about assessments, systematically tracking outcomes, altering plans of care based on assessments, giving feedback on outcomes, and using assessments to choose services. Regarding between-group differences, facilitators in the EHR group reported sending reminders to a significantly smaller proportion of families compared to those in the SAU group. Facilitator reports did not differ by treatment group for the remaining subscales.

## Discussion

### Principal Results

Research on the use of EHR systems in behavioral healthcare has lagged behind research in general healthcare, resulting in a dearth of empirical guidance around issues such as software design and the impact of EHR adoption on services. The current study attempted to fill gaps in the research base by asking whether care coordinators serving children and youth with complex behavioral health needs who were randomly assigned to use an EHR would demonstrate differences in service processes and service quality compared to providers using paper records. Results indicated that there were few such impacts. No between-group differences were found for fidelity to the Wraparound practice model, an overall working alliance among practitioners and families, Wraparound team climate, parent satisfaction with care, or practitioner satisfaction with services.

At the same time, practitioners in the EHR group reported spending significantly more time reviewing and applying client progress data in supervision, and significantly less time on administrative tasks. This finding provides support to the proposal that use of EHR systems can facilitate greater attention to client progress and subsequent problem solving and is consistent with prior research indicating that digital feedback technologies can effectively support assessment-related provider behavior change [63,64]. This is an encouraging result given that “treating to target” is a commonly-cited principle of effective behavioral healthcare, and has been found to account for substantial variance in positive outcomes [65]. Also, results from the CAPER found that facilitators in the EHR group were significantly less likely to send reminders to enrolled clients. Given that reminders around meetings and appointments can be automatically undertaken by the EHR, it may be that EHR use reduced the need for facilitators to do these tasks manually, potentially freeing time for other tasks.

Results from the CAPER also showed significant increases among facilitators in both groups for collecting and using assessment and outcomes data, altering plans of care based on assessments, and using assessments to choose services. Although between-group effects were not found, leaders in the two agencies suggested that these significant increases may have been a result of the EHR influencing supervisor behavior with facilitators in both groups, and peer influence among practitioners within the agency.

Not all significant results supported positive impacts of the EHR. First, there was a pattern of poorer scores for the EHR group on the WAI, including a trend toward significance (*P*=.10) on the subscale focused on agreement on tasks to achieve identified goals. While such findings may have been spurious given the number of statistical tests conducted, they also may indicate that the time and effort needed to integrate a new EHR into workflow compromised engagement and alliance between EHR-assigned facilitators and families.

Second, perceptions of psychometric quality of standardized assessments improved over time for the SAU group but not the EHR group, resulting in a significant between-group difference for this subscale. Although a subtle effect, it may be that these provider organizations’ increased attention to use of measurement and measurement scales was received more enthusiastically among staff waiting to be trained on the EHR than the initial adopters, who were exposed to the day-to-day realities and challenges of a rapid training and implementation process on the EHR, as well as shifts in how supervision was conducted.

All the above findings must be interpreted within the context of practitioners’ perceptions of usability and acceptability of this particular software package. Results from surveys suggested that staff perceived the software to be reasonably well-aligned with the Wraparound practice model and the day-to-day workflow of facilitators. Mean ratings of overall usability were, however, lower, scoring in the “marginal” range on the SUS. Qualitative feedback from staff assigned to the EHR condition indicates that at the launch of the study, the EHR had some functionality issues (eg, frequent timing out, multiple clicks required to execute simple but frequently required tasks) that compromised its ease of use. Although the study (and the larger federally-funded project within which the study was conducted) allowed such issues to be identified and addressed by programmers, usability issues at the outset may have compromised the capacity for the EHR to achieve its full proposed impact.

Future studies would benefit from an examination of the impact of usability of EHR systems (or impact across multiple stages of development or implementation of a single system) on outcomes. It is important to note that successful application of EHRs—and HIT in general—requires strategic implementation supports to be successfully applied [66-68]. The rapid timeframe for the current study meant that EHR training and initiation of youth/family study enrollment happened very quickly and with less development of readiness and local implementation support than may have been ideal. Although service quality and fidelity were not assessed, it is worth noting that six months after initiation of the current study, the larger of the two provider organizations introduced a refined variant of the EHR in another site with all its staff at once and with local staff who had participated in the current study leading the roll-out and supporting implementation. Mean SUS scores for this cohort of facilitators was 63.7, nearly 10 points higher than for the EHR-assigned group in the current study.

### Limitations

The current study has several major limitations. It focused only on short-term (4 months) outcomes, and these were limited to the provider, workflow, and service variables. The actual impact on outcomes such as residential placement, symptoms, and functioning and family outcomes such as family functioning or caregiver strain were not assessed. The sample size was small, and over one-third of the initial sample of youth/caregivers was lost to follow-up, limiting our ability to detect significant differences. As described above, randomization at the site or supervisor level was not possible, meaning that between-group contamination (eg, in areas such as supervision style or activities or use of standardized assessment) may have occurred. This may also have compromised the study’s ability to detect impacts of the EHR.

Finally, as described above, the funding mechanism for the study only provided one year for development and refinement of the system followed by a single year to undertake a randomized pilot study, hindering the usability and implementation of the EHR. Although research and experience suggest that practitioner perceptions of EHR system usability and provision of training and implementation support are often poor in behavioral health, this situation may reduce the generalizability of results. Conducting the study in the context of Wraparound facilitation, which consists of a relatively unique set of practice activities, also may limit generalizability to other service types.

### Implications

Along with other subtypes of HIT, EHR systems have been increasingly proposed as a method to support service quality implementation support, functioning as a practitioner-facing implementation strategy that can help organize plans of care, provide reminders, and structure workflow and supervision [69-71]. Results of the current study suggest that even when implemented under unideal circumstances (eg, a randomized study within an organization), the introduction of an EHR may indeed facilitate measurable and beneficial shifts in practice, such as greater attention to measurement-based care. At the same time, results suggest that EHRs may give rise—at least initially—to measurable, if subtle, negative impacts, such as less capacity for practitioners to nurture engagement and alliance.

These findings align with findings from other studies [15,72] that work tasks can be influenced positively by EHR adoption. At the same time, this research also supports conclusions by other researchers that productivity, and presumably quality of care, may decrease after initial implementation of an EHR, primarily as a result of the implementation effort typically required, and that no less than one month may be required after transition to a new EHR before practitioners return to baseline productivity [18,73]. This is also consistent with research on the implementation of new practices in general, where an initial decrease in competence might be expected before providers building mastery of the innovation [74].

Research is needed that provides more rigorous tests of these associations, and that can unpack the underlying causes. In this study, for example, it is unclear whether greater benefits of EHR implementation would have been found had the system featured greater usability at the outset, rather than still undergoing improvement during the study. Fleming and colleagues, for example, found that it took 12 months for overall productivity to rebound to baseline levels after installation of an EHR [18]. Although logistical and methodological challenges may arise, researchers conducting more robust controlled tests of EHR systems in the future may be advised to wait up to one-year post-implementation before assessing impacts.

Similarly, it is unclear whether more robust implementation support (and agency-wide versus partial implementation) may have resulted in different outcomes. Future studies may focus on these issues by using “hybrid trial” approaches that simultaneously consider—or experimentally manipulate—EHR usability and contextual fit, implementation strategies, and outcomes [75]. Given the level of prioritization of HIT generally and EHR specifically in behavioral healthcare—and the number of system resources and human capital being invested in these technologies—continued expansion of the research base on these topics would seem to be a critically important investment.

## Appendix

Multimedia Appendix 1 CONSORT‐EHEALTH checklist (V.1.6.1).

## Abbreviations

ASA: Attitudes toward Standardized Assessment Scale
BSPC: Brief Supervision Practice Checklist-Adapted
CAPER: Current Assessment Practice Evaluation-Revised
CSQ: Client Satisfaction Questionnaire
EHR: electronic health record
HIT: health information technology
HITECH Act: Health Information Technology for Economic and Clinical Health
ICC: intraclass correlation coefficient
SAAS: System Acceptability and Appropriateness Scale
SAU: services as usual
SPQ: Supervision Process Questionnaire
SUS: System Usability Scale
TCI: Team Climate Inventory, Short Version
WAI: Working Alliance Inventory
WFI-EZ: Wraparound Fidelity Index, Brief Version
